# Contribution of SLC30A8 variants to the risk of type 2 diabetes in a multi-ethnic population: a case control study

**DOI:** 10.1186/1472-6823-14-2

**Published:** 2014-01-06

**Authors:** Sameer D Salem, Riyadh Saif-Ali, Ikram S Ismail, Zaid Al-Hamodi, Sekaran Muniandy

**Affiliations:** 1 Department of Molecular Medicine, Faculty of Medicine, University of Malaya, 50603 Kuala Lumpur, Malaysia; 2 Department of Biochemistry, Faculty of Medicine, Sana’a University, Sana’a, Yemen; 3 Department of Medicine, Faculty of Medicine, University of Malaya Medical Centre, University of Malaya, 50603 Kuala Lumpur, Malaysia

**Keywords:** T2D, GADA negative diabetes, SLC30A8, Haplotypes, Alternative variants

## Abstract

**Background:**

Several studies have shown the association of solute carrier family 30 (zinc transporter) member 8 (SLC30A8) rs13266634 with type 2 diabetes (T2D). However, the association of alternative variants and haplotypes of SLC30A8 with T2D have not been studied in different populations. The aim of this study is to assess the association of the alternative SLC30A8 variants, rs7002176 and rs1995222 as well as the most common variant, rs13266634 and haplotypes with glutamic acid decarboxylase antibodies (GADA) negative diabetes in Malaysian subjects.

**Methods:**

Single nucleotide polymorphisms (SNPs) of SLC30A8; rs7002176, rs1995222 and rs13266634 were genotyped in 1140 T2D and 973 non-diabetic control subjects. Of these, 33 GADA positive diabetic subjects and 353 metabolic syndrome (MetS) subjects were excluded from subsequent analysis.

**Results:**

The recessive genetic model controlled for age, race, gender and BMI shows that the alternative SLC30A8 variant, rs1995222 is associated with GADA negative diabetes (OR = 1.29, P = 0.02) in Malaysian subjects. The most common variant, rs13266634 is also associated with GADA negative diabetes (OR = 1.45, P = 0.001). This association is more pronounced among Malaysian Indians (OR = 1.93, P = 0.001). Moreover, the CG haplotype and CG-CG diplotype have been equally associated with increased diabetic risk (OR = 1.67, P = 8.6 × 10^-5^).

**Conclusions:**

SLC30A8 SNPs and haplotypes are associated with GADA negative diabetes in Malaysian subjects, and this association is markedly higher among Malaysian Indian subjects.

## Background

Type 2 diabetes (T2D) is a serious public health problem with its prevalence rapidly increases globally. In the next two decades, Asian countries will be hit hardest, particularly China and India where the diabetic populations will more than double [1-3]. In Malaysia, more than 2.1 million of the adult population have diabetes (11.7%) [3]. T2D is a complicated metabolic disorder, characterized by insulin resistance and/or pancreatic β-cell dysfunction resulting from both genetic and environmental factors [2,4,5]. Among T2D patients, latent autoimmune diabetes of adults (LADA) occurs in 2-12% of individuals [6-9]. LADA can be distinguished by the presence glutamic acid decarboxylase antibodies (GADA) in adult diabetic patients who clinically are similar to T2D subjects at diagnosis [7,10-12] and are insulin independent for at least in the first six months [13].

The interaction of a stable genetic background with the rapidly changing environment has resulted in rapid changes in the prevalence of T2D observed over recent decades [14]. Insulin resistance has been proposed to be a major driver of progression to T2D. However, most of the validated genetic variants are involved in β-cell function. The genome-wide association studies (GWAS) approach has dramatically increased the number of T2D susceptibility loci, expanding the list from five loci in 2007 to more than 60 loci in 2012. The association to T2D of more than 20 newly reported loci in Asians [15,16] and Europeans [17] needs to be studied in other populations as well. A consistent association of T2D risk with variants of the pancreatic β-cell–specific zinc transporter gene SLC30A8 has been discovered in European subjects [18-23]. This has been also reported in multi-ethnic case-control studies, including Asian [2,24-32], Arabian [33], European [17] and American populations [34]. The link between impaired β-cell function and Zn transport activity by SLC30A8 has been reported in several studies [35-39]. The consensus is that SLC30A8 is crucial for insulin processing and secretion, and the major contribution of the SLC30A8 SNPs to T2D is mediated through defects in insulin secretion rather than action. The SLC30A8 gene encodes the ZnT-8 zinc transporter, which is exclusively expressed in pancreatic β-cells and co-localized with insulin-containing secretory granules [40,41]. SLC30A8 variants impair islet ZnT8 expression, insulin secretion, or glucose homeostasis [39,42,43]. In addition, these variants are associated with the production of a less active zinc transporter protein, suggesting less efficiency of zinc accumulation and insulin crystallization [44]. ZnT-8 is thought to be a key protein for insulin secretion by regulating the homeostasis of zinc, which is an essential metal ion for insulin storage and secretion into intracellular vesicles [45,46].

Studies on the association of variants of the SLC30A8 gene with T2D have been concerned with the most common SNP rs13266634. However, the association of alternative SLC30A8 SNPs and haplotypes with T2D has not been studied in different populations. The aim of this investigation was to study the association of alternative SLC30A8 SNPs (rs7002176, rs1995222) and haplotypes with GADA negative diabetes, and further to replicate the association of the most common variant, rs13266634 in Malaysian subjects.

## Methods

1 Subjects and data collection

This hospital-based case-control study was been conducted at the University Malaya Medical Centre (UMMC), Kuala Lumpur. Patients previously diagnosed with T2D (FPG ≥ 7.0 mmol/l), who attended the UMMC for treatment were invited to participate in this study (the case group). For the control group, subjects who were enrolled for general health screening at UMMC (FPG ≤ 6.1 mmol/l) were approached to participate in this study. GADA positive diabetic patients and metabolic syndrome **(**MetS) subjects were excluded from this study. Data collection for this study took place between 2009 and 2011. Details regarding the study design, including subjects, data collection, demographic parameters, biochemical analysis and quality control have been reported previously [47]. This study has been approved by the Medical Ethics Committee of University Malaya Medical Centre. A written informed consent was obtained from each participant in the study.

2 SNPs selection and genotyping

SLC30A8 SNPs, rs7002176, rs1995222 and rs13266634 were been selected based on previous studies [18,48]. Genomic DNA was isolated from peripheral blood leukocytes using Wizard® Genomic DNA Purification Kit (Promega Corporation, Madison, WI, USA) according to the manufacturer’s protocol. The SNPs were genotyped by pre-designed Taqman genotype assays (C_29002970_10, C_1421536_10 and C_357888_10 respectively, Applied Biosystems Inc, Foster City, USA) according to the manufacturer’s protocol using StepOnePlus Real-Time PCR system (Applied Biosystems Inc, Foster City, USA). No-template controls (NTCs) were included together with samples in each batch. The genotype call rates for SNPs, rs7002176, rs1995222 and rs13266634 were 98.5% (1130 diabetes; 951 non-diabetes), 97.1% (1108 diabetes; 943 non-diabetes) and 99.1% (1136 diabetes; 960 non-diabetes) respectively. The concordance rate, based on blind duplicate comparisons (10% of the samples that were blindly re-genotyped) was 100%.

3 Statistical analysis

Deviation of genotypes from Hardy-Weinberg Equilibrium was assessed with the DeFinetti program (http://ihg.gsf.de/cgi-bin/hw/hwa1.pl from the Institute of Human Genetics). The linkage disequilibrium (LD) between SNPs and the construction of haplotypes and diplotypes of related SNPs were performed with SNP & Variation Suite v7.x program (Golden Helix, Bozeman, MT, USA). Social Package of Statistical Science (SPSS version 11.5, LEAD Technologies; Inc. USA) was used to study the associations of SNPs of SLC30A8 using recessive, dominant and additive genetic models with T2D. These associations were evaluated by logistic regression analysis controlled for age, gender and body mass index. Significance was inferred when P < 0.05.

## Results and discussion

The study included 1140 T2D and 973 non-diabetic control subjects. GADA positive diabetic subjects are classified as LADA since the genetic causes of this class of diabetes are similar to type 1 diabetes. To minimize variation among the diabetic group, the 33 GADA positive diabetic subjects were excluded from the study. Application of the new metabolic syndrome (MetS) criteria [49] on non-diabetic control subjects revealed that 353 subjects had MetS. Metabolic syndrome is a strong risk factor for diabetes which may affect the association study and Hardy-Weinberg Equilibrium. Hence, subjects in the control group with MetS were excluded from subsequent analysis. The metabolic and diabetic parameters were been significantly different between the target and control groups (Table 1).

1 Association of SLC30A8 SNPs with GADA negative diabetes

SNPs of SLC30A8, rs7002176, rs1995222 and rs13266634 showed no deviation from Hardy-Weinberg Equilibrium in the control group (P-value = 0.36, 0.54, 0.40 respectively). The alternative SLC30A8 SNPs; rs7002176 and rs1995222 were evaluated in this study. The logistic regression model (adjusted for age, race, gender and BMI) for rs1995222 showed association (recessive genetic model, OR = 1.29; P = 0.02) with GADA negative diabetes in Malaysian subjects. This finding is in contrast with findings in Pima Indian [48]. However, the SNP rs7002176 showed no association with GADA negative diabetes, a finding which is in agreement with Rong *et al.*[48]. The finding of non-association might be explained by the differences in genetic background, genetic model used and ethnicities between the populations [17,26] (Table 2).

The most common variant, rs13266634 showed significant association with GADA negative diabetes in Malaysian subjects (recessive genetic model, OR = 1.45, P = 0.001). This finding is in agreement with previous studies in various Asian populations [2,24-32]. However, the association of this SNP with GADA was different for subjects of the three main Malaysian races (Malay, Chinese and Indian). The rs13266634 SNP was strongly associated with GADA negative diabetes among Malaysian Indian subjects (recessive genetic model, OR = 1.93, P = 0.001), but not with Malaysian Chinese and Malay subjects (Table 2). Tan *et al.*[30] have shown similar association with diabetes among Singaporean Malay subjects, whereas, their finding among Singaporean Chinese and Indian subjects have shown no association. This discrepancy in association of this SNP with GADA among Asian populations might be due to the small sample size used in the analysis. In addition, the environmental risk profile, lifestyle, body composition and linkage disequilibrium patterns might be involved. Likewise, the association between rs13266634 in the SLC30A8 gene locus and susceptibility to T2D has been demonstrated in Caucasian populations [19,20,22,23]. The rs13266634 SNP is a nonsynonymous Arg^325^ → Trp^325^ variant in the zinc transporter SLC30A8 [19,50]. Since SLC30A8 encodes a zinc transporter expressed solely in the secretory vesicles of β-cells, and is implicated in the final stages of insulin biosynthesis involving co-crystallization with zinc [18], its association with T2D is to be expected.

Previous studies had suggested that the major contribution of the SLC30A8 SNPs to T2D was mediated through defects in insulin secretion rather than action [35]. However, neither of these SNPs showed an association with HOMA-β nor HOMA-IR (Additional file 1: Table S1). Similar outcomes have been reported in other studies [2,26]. These conflicting results on the role of SLC30A8 in insulin secretion might be explained by the different genetic background between different populations. Moreover, other gene interactions, that may also have contributed in the metabolism and regulation of insulin activity [46].

2 Association of SLC30A8 haplotypes and diplotypes with GADA negative diabetes

**Figure 1:**
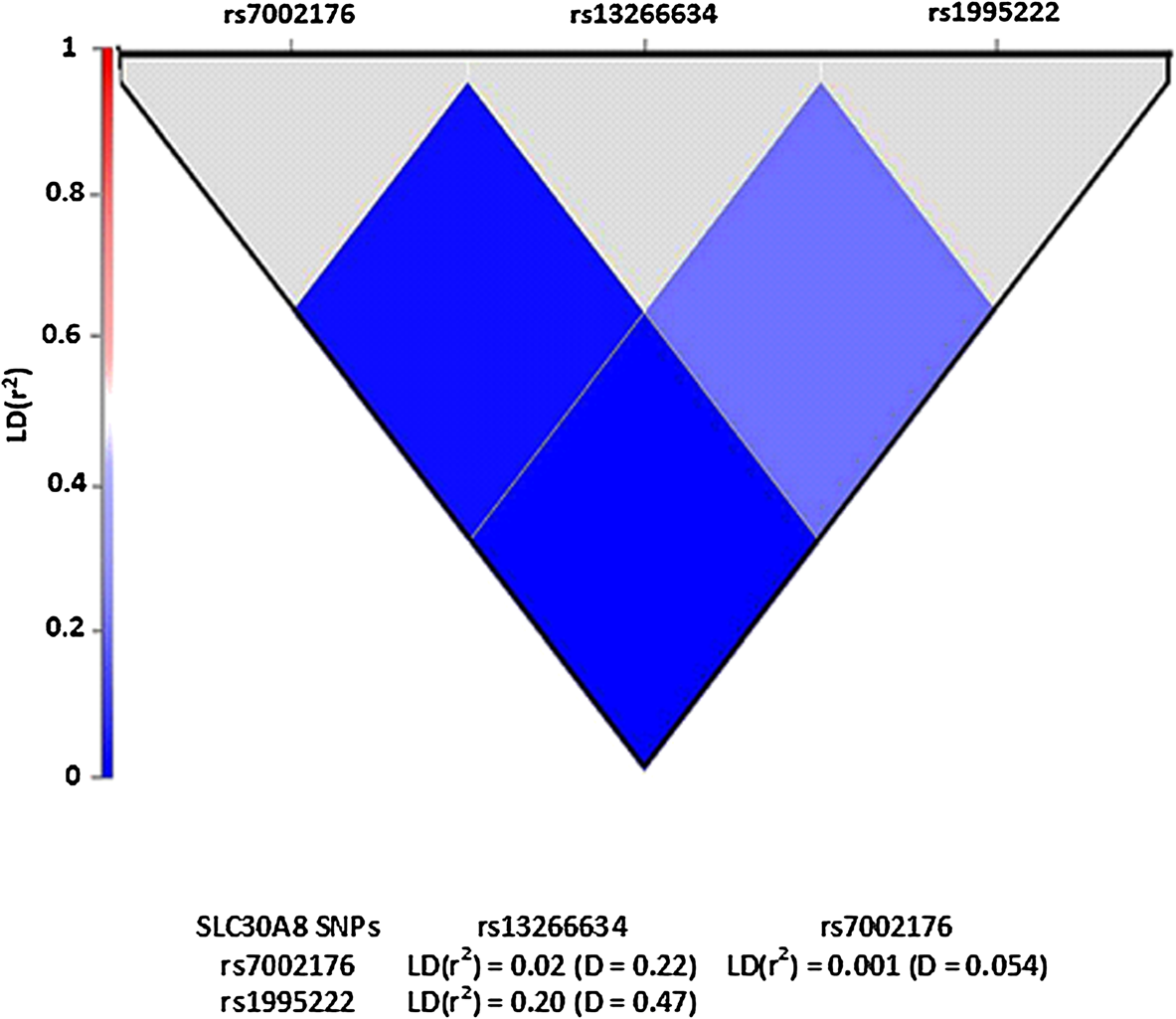
Pairwise linkage disequilibrium among SLC30A8 SNPs in Malaysian subjects.

Two-SNP haplotype and diplotype blocks (rs1995222 and rs13266634) with significant LD were identified (Figure 1). There was a significant linkage between SNPs rs13266634 and rs1995222 (r^2^ = 0.20), although, the distance between the two SNPs is approximately 45 kb. This finding is similar to that reported in Europeans (r^2^ = 0.19). However, SNP rs13266634 is near to SNP rs7002176, the distance between the two SNPs is approximately 2.7 kb, but there is no significant linkage between them. The haplotypes and diplotypes with frequency < 2% of the combined races were been excluded from subsequent analysis. The logistic regression model (adjusted for age, race, gender and BMI) showed that the haplotype CG and the diplotype CG-CG (containing the risk alleles of the SNPs) are equal risk factors for T2D in the combined races (OR = 1.67, P = 8.6 × 10^-5^), and this risk is higher in diabetic Indian subjects (OR = 1.93, P = 0.001) (Table 3). The haplotype (CG) and diplotype (CG-CG) showed a stronger association with GADA negative diabetes than single individual SNPs. This strength could be attributed to the possible epistatic interaction between these SNPs in determining overall risk of T2D. The haplotypes and diplotypes are not associated with GADA negative diabetes in Malay and Chinese subjects except the diplotype AC-AT is associated with protection against diabetes in Malay subjects (OR = 0.26, P = 0.0002) (Additional file 1: Table S2). The frequencies of haplotype GT and diplotype CG-GT are higher in Indian subjects without diabetes compared to Indian subjects with diabetes (OR = 0.51; 0.48, P = 0.008; 0.005, respectively) (Table 3). This study is a hospital-based, and the sampling method is non-probability. Thus, the sampling for this study has limited its generalization to the whole Malaysian population. Sub-grouping of subjects according to races resulted in small sample size, which likely gives insufficient power to provide an evidence for association within the ethnic subgroups.

**Table 1:** Demographic and biochemical characterizations of participants

**Demography and biochemical profiles**		**Normal control**	**GADA negative diabetes**
		**n = 620**	**n = 1107**
Gender %	Male/female	61.6/38.4	67.6/32.4
Races %	Malay	41.0	38.4
	Chinese	32.9	26.5
	Indian	26.1	35.1
Age (years)		49.5(48.6–50.3)	51.3(50.8–51.9)
P*-*value			***0.001***
Body mass index (kg/m^2^)		24.6(24.3–25.0)	27.4(27.1–27.7)
P*-*value			***<0.001***
Waist circumference (cm)		85.2(84.2–86.2)	96.1(95.4–96.8)
P*-*value			***<0.001***
Systolic blood pressure (mmHg)		128(127–130)	134(133–135)
P*-*value			***<0.001***
Diastolic blood pressure (mmHg)		80(79–80)	81(80–82)
P*-*value			***0.005***
Fasting plasma glucose (mmol/l)		4.98(4.94–5.02)	7.89(7.73–8.05)
P*-*value			***<0.001***
Fasting plasma insulin (pmol/l)		59.65(56.35–63.15)	98.15(94.08–102.39)
P*-*value			***<0.001***
HOMA-β		111.35(107.19–115.67)	70.84(67.41–74.44)
P*-*value			***<0.001***
HOMA-IR		1.27(1.20–1.35)	2.45(2.34–2.55)
P*-*value			***<0.001***
High density lipoprotein (mmol/l)		1.34(1.32–1.37)	1.09(1.07–1.10)
P*-*value			***<0.001***
Triglyceride (mmol/l)		1.13(1.09-1.17)	1.58(1.53–1.63)
P*-*value			***<0.001***

The results presented represent geometric means (95% confidence interval of the mean), which evaluated by ANOVA. Bolded values are significant. GADA: glutamic acid decarboxylase antibodies.

**Table 2:** Association of SLC30A8 polymorphism with GADA negative diabetes among Malaysian subjects and ethnic groups

**SLC30A8 SNPs**	**Control**		**GADA negative diabetes**		**Recessive**	**Dominant**	**Additive**
					**OR (95% CI)**		
	**Freq.**	**11/12/22**	**Freq.**	**11/12/22**	**P-value**		
**rs7002176 (A > T)**							
Combined^*^	0.42	113/284/208	0.45	222/541/335	1.12(0.85–1.46) 0.43	1.17(0.93–1.47) 0.18	1.11(0.95–1.29) 0.19
Malay^#^	0.37	35/115/100	0.42	79/191/150	1.61(1.00–2.59) 0.05	1.16(0.80–1.67) 0.43	1.23(0.96–1.57) 0.11
Chinese^#^	0.48	46/98/55	0.47	56/160/75	0.70(0.43–1.14) 0.15	1.06(0.67–1.65) 0.81	0.89(0.67–1.19) 0.43
Indian^#^	0.43	32/71/53	0.47	87/190/110	1.12(0.70–1.78) 0.65	1.32(0.88–1.99) 0.18	1.22(0.93–1.60) 0.15
**rs1995222 (A < G)**							
Combined^*^	0.63	238/273/87	0.67	498/440/138	1.29(1.03–1.62) ***0.02***	1.17(0.85–1.60) 0.34	1.18(1.01–1.39) ***0.039***
Malay^#^	0.60	88/116/40	0.64	167/189/54	1.22(0.85–1.77) 0.28	1.55(0.93–2.58) 0.10	1.24(0.95–1.61) 0.11
Chinese^#^	0.54	57/97/43	0.54	87/133/66	1.16(0.75–1.79) 0.51	0.97(0.60–1.56) 0.89	1.05(0.80–1.38) 0.73
Indian^#^	0.78	93/60/4	0.80	244/118/18	1.26(0.85–1.87) 0.25	0.27(0.06–1.22) 0.09	1.11(0.79–1.56) 0.53
**rs13266634 (C > T)**							
Combined^*^	0.60	223/284/105	0.65	506/415/181	1.45(1.16–1.81) ***0.001***	0.99(0.75–1.32) 0.96	1.18(1.01–1.37) ***0.03***
Malay^#^	0.58	84/127/42	0.60	158/192/73	1.16(0.80–1.66) 0.44	0.85(0.53–1.35) 0.49	1.02(0.79–1.30) 0.90
Chinese^#^	0.50	56/90/56	0.49	85/115/91	1.14(0.74–1.76) 0.55	0.93(0.61–1.44) 0.76	1.03(0.79–1.33) 0.84
Indian^#^	0.74	83/67/7	0.82	263/108/17	1.93(1.31–2.85) ***0.001***	0.81(0.31–2.14) 0.68	1.58(1.14–2.17) ***0.006***

Risk allele frequency (Freq.) and genotype counts in individuals with GADA negative diabetes and control subjects. 11, homozygous of major allele; 12, heterozygous; 22, homozygous of minor allele. GADA, glutamic acid decarboxylase antibodies. Risk allele is denoted in boldface. Bolded values are significant. *Controlled for age, race, gender and BMI. #Controlled for age, gender and BMI. The outliers (studentized residual is greater than 2.0 or less than −2.0) were excluded.

**Table 3:** Association of SLC30A8 common haplotypes and diplotypes with GADA negative diabetes among combined races and Indian subjects

**rs13266634, rs1995222**	**Combined races**				**Indians**			
	**Frequency**		**P-value**	**OR (95% CI)**	**Frequency**		**P-value**	**OR (95% CI)**
	**Control**	**GADA negative diabetes**			**Control**	**GADA negative diabetes**		
**Haplotypes**								
AT	0.44	0.39	0.10	0.83(0.67–1.03)	0.23	0.20	0.28	0.78(0.50–1.23)
CG	0.21	0.32	***8.6 × 10***^***-5***^	1.67(1.29–2.15)	0.36	0.51	***0.001***	1.93(1.31–2.85)
GT	0.17	0.14	0.08	0.77(0.58–1.03)	0.22	0.12	***0.008***	0.51(0.31–0.84)
AC	0.14	0.13	0.59	0.92(0.67–1.25)	0.15	0.15	0.91	0.97(0.57–1.65)
**Diplotypes**								
CG-CG	0.21	0.32	***8.6 × 10***^***-5***^	1.67(1.29–2.15)	0.36	0.51	***0.001***	1.93(1.31–2.85)
CG-AT	0.24	0.22	0.36	0.89(0.69–1.14)	0.20	0.16	0.15	0.70(0.43–1.13)
CG-GT	0.14	0.11	0.09	0.76(0.55–1.05)	0.20	0.11	***0.005***	0.48(0.29–0.80)
CG-AC	0.12	0.11	0.62	0.92(0.67–1.27)	0.14	0.13	0.94	1.02(0.58–1.79)
GT-AT	0.08	0.07	0.62	0.90(0.60–1.36)	0.02	0.01	0.30	0.48(0.12–1.91)
AC-AT	0.07	0.04	***0.001***	0.43(0.27–0.70)	0.006	0.01	NA	NA

Controlled for age, gender, race and BMI. The outliers (studentized residual is greater than 2.0 or less than−2.0) were excluded. GADA, glutamic acid decarboxylase antibodies.

## Conclusions

The most common SLC30A8 variant, rs13266634 is associated with GADA negative diabetes in Malaysian subjects, and this association is more pronounced among Malaysian Indian subjects. In addition, the alternative SLC30A8 variant, rs1995222 is significantly linked with rs13266634 (r^2^ = 0.2) and shows a mild association with GADA negative diabetes. The risk of these SNPs is strengthened by the haplotypes and diplotypes containing the SNPs risk alleles.

## Abbreviations

SLC30A8: Solute carrier family 30 (zinc transporter), member 8; T2D: Type 2 diabetes; SNP: Single nucleotide polymorphisms; GADA: Glutamic acid decarboxylase antibodies; OR: Odds ratio; GWAS: Genome-wide association studies; UMMC: University Malaya Medical Centre; NTC: No template controls; LD: Linkage disequilibrium; MetS: Metabolic syndrome; BMI: Body mass index; HOMA-β: Homeostasis model assessment of β-cell function; HOMA-IR: Homeostasis model assessment of insulin resistance.

## Competing interests

The authors declare that they have no competing interests.

## Authors’ contributions

SDS conceived the design of the study, collected the data, performed the experiments, statistical analyses and drafted the manuscript. RSA participated in the design of the study, statistical analyses and drafted the manuscript. ISI and ZA participated in collection of data. SM participated in the design of the study and reviewed the manuscript. All authors read and approved the final manuscript.

## Pre-publication history

The pre-publication history for this paper can be accessed here:

http://www.biomedcentral.com/1472-6823/14/2/prepub

## Supplementary Material

Additional file 1: Table S1 Impact of SLC30A8 SNPs, haplotypes and diplotypes on beta-cell function (HOMA-β) and insulin resistance (HOMA-IR) in normal Malaysian subjects. **Table S2.** Association of SLC30A8 common haplotypes and diplotypes with GADA negative diabetes among Malaysian Malay and Chines subjects. Click here for file
