# Supplementary material for: Contribution of SLC30A8 variants to the risk of type 2 diabetes in a multi-ethnic population: a case control study
Source: BMC Endocr Disord. 2014 Jan 6;14:2. doi: 10.1186/1472-6823-14-2 (PMC3893602; doi:10.1186/1472-6823-14-2)
Supplement: Additional file 1: Table S1 — Impact of SLC30A8 SNPs, haplotypes and diplotypes on beta-cell function (HOMA-β) and insulin resistance (HOMA-IR) in normal Malaysian subjects. Table S2. Association of SLC30A8 common haplotypes and diplotypes with GADA negative diabetes among Malaysian Malay and Chines subjects. [file 1472-6823-14-2-S1.docx]

# Additional files

**Table S1.** Impact of SLC30A8 SNPs, haplotypes and diplotypes on beta-cell function (HOMA-β) and insulin resistance (HOMA-IR) in normal Malaysian subjects.

| **SLC30A8 SNPs, Haplotypes and Diplotypes** | **HOMA-β**  Mean (CI) | Parameter  estimate  P-Value | Univariate  P-Value | **HOMA-IR**  Mean (CI) | Parameter  estimate  P-Value | Univariate  P-Value |
| --- | --- | --- | --- | --- | --- | --- |
| **rs7002176** | | | | | | |
| A-A (n=113) | 109(101-118) | 0.48 | 0.77 | 1.28(1.15-1.43) | 0.96 | 0.97 |
| A-T (n=284) | 112(107-118) | 0.86 |  | 1.27(1.19-1.36) | 0.80 |  |
| T-T (n=208) Ref | 113(107-119) |  |  | 1.29(1.19-1.39) |  |  |
| **rs13266634** | | | | | | |
| C-C (n=223) | 111(105-117) | 0.89 | 0.88 | 1.30(1.20-1.40) | 0.32 | 0.60 |
| C-T (n=284) | 112(107-118) | 0.66 |  | 1.28(1.20-1.37) | 0.40 |  |
| T-T (n=105) Ref | 110(102-119) |  |  | 1.21(1.08-1.36) |  |  |
| **rs1995222** | | | | | | |
| G-G (n=238) | 114(109-120) | 0.07 | 0.18 | 1.33(1.24-1.44) | ***0.028*** | 0.08 |
| A-G (n=273) | 112(107-118) | 0.13 |  | 1.30(1.21-1.39) | 0.059 |  |
| A-A (n=87) Ref. | 104(96-113) |  |  | 1.13(1.01-1.28) |  |  |
| **Haplotypes** | | | | | | |
| AC (n=86) | 104(95-113) | 0.12 | 0.21 | 1.21(1.07-1.37) | 0.52 | 0.29 |
| GT (n=106) | 111(102-120) | 0.78 |  | 1.25(1.12-1.41) | 0.86 |  |
| CG (n=132) | 117(109-125) | 0.35 |  | 1.39(1.26-1.54) | 0.13 |  |
| AT (n=273) Ref. | 112(107-118) |  |  | 1.27(1.18-1.36) |  |  |
| **Diplotypes** | | | | | | |
| CG-AC (n=76) | 105(96-116) | 0.82 | 0.23 | 1.23(1.07-1.40) | 0.33 | ***0.045*** |
| CG-GT (n=85) | 112(102-122) | 0.57 |  | 1.26(1.12-1.43) | 0.20 |  |
| CG-CG (n=132) | 118(110-126) | 0.19 |  | 1.41(1.28-1.56) | ***0.014*** |  |
| CG-AT (n=149) | 118(110-126) | 0.17 |  | 1.41(1.29-1.55) | ***0.011*** |  |
| AC-AT (n=45) Ref. | 107(95-121) |  |  | 1.10(0.93-1.30) |  |  |

HOMA-β (β-cell function) and HOMA-IR (insulin resistance) were log-transformed before analysis, and the data were presented as geometric means and 95% confidence interval of the mean which evaluated by univariate (General Linear Model). Ref, reference, the protective genotype, haplotypes and diplotypes were selected to be a reference for the comparison; CI, confidence interval.

**Table S2.** Association of SLC30A8 common haplotypes and diplotypes with GADA negative diabetes among Malaysian Malay and Chines subjects.

| **rs13266634,**  **rs1995222** | | **Control** | | | **GADA negative diabetes** | |
| --- | --- | --- | --- | --- | --- | --- |
|  |  | **Malay (n=254)** | **Chinese (n=204)** | | **Malay (n=425)** | **Chinese (n=293)** |
| **Haplotypes** | | | | |  | |
| AT | Freq. | 119(0.47) | 116(0.57) | | 191(0.45) | 165(0.56) |
|  |  | OR (95% CI) | | | 0.94(0.66-1.33) | 0.88(0.59-1.31) |
|  |  | P-Value | | | 0.71 | 0.52 |
| CG | Freq. | 44(0.17) | 30(0.15) | | 103(0.24) | 49(0.17) |
|  |  | OR (95% CI) | | | 1.44(0.93-2.24) | 1.22(0.71-2.08) |
|  |  | P-Value | | | 0.10 | 0.47 |
| GT | Freq. | 44(0.17) | 27(0.13) | | 64(0.15) | 38(0.13) |
|  |  | OR (95% CI) | | | 0.91(0.57-1.47) | 1.02(0.56-1.84) |
|  |  | P-Value | | | 0.71 | 0.95 |
| AC | Freq. | 37 (0.15) | 24(0.12) | | 52(0.12) | 34(0.12) |
|  |  | OR (95% CI) | | | 0.80(0.48-1.31) | 1.01(0.55-1.84) |
|  |  | P-Value | | | 0.37 | 0.97 |
| **Diplotypes** | | | | |  | |
| CG-CG | Freq. | 44(0.17) | 30(0.15) | | 103(0.24) | 49(0.17) |
|  |  | OR (95% CI) | | | 1.44(0.93-2.24) | 1.22(0.71-2.08) |
|  |  | P-Value | | | 0.10 | 0.47 |
| CG-AT | Freq. | 64(0.25) | 52(0.25) | | 112(0.26) | 68(0.23) |
|  |  | OR (95% CI) | | | 1.11(0.75-1.67) | 0.90(0.57-1.41) |
|  |  | P-Value | | | 0.60 | 0.64 |
| CG-GT | Freq. | 31(0.12) | 21(0.10) | | 52(0.12) | 25(0.09) |
|  |  | OR (95% CI) | | | 1.03(0.60-1.76) | 1.01(0.51-2.00) |
|  |  | P-Value | | | 0.92 | 0.99 |
| CG-AC | Freq. | 35(0.14) | 19(0.09) | | 43(0.10) | 32(0.11) |
|  |  | OR (95% CI) | | | 0.69(0.41-1.15) | 1.20(0.63-2.28) |
|  |  | P-Value | | | 0.16 | 0.58 |
| GT-AT | Freq. | 17(0.07) | 26(0.13) | | 34(0.08) | 33(0.11) |
|  |  | OR (95% CI) | | | 1.57(0.79-3.09) | 0.71(0.39-1.31) |
|  |  | P-Value | | | 0.20 | 0.28 |
| AC-AT | Freq. | 28 (0.11) | | 16(0.08) | 20(0.05) | 21(0.07) |
|  |  | OR (95% CI) | | | 0.26(0.13-0.53) | 0.67(0.31-1.46) |
|  |  | P-Value | | | ***0.0002*** | 0.31 |

Controlled for age, gender and BMI. The outliers (studentized residual ≥ 2.0 or ≤ −2.0) were excluded. GADA, glutamic acid decarboxylase antibodies.
